# Diagnostic turnaround time and antimicrobial use in suspected central nervous system infection: a regional Australian cohort study

**DOI:** 10.1017/ash.2026.10393

**Published:** 2026-05-12

**Authors:** Semun Stewart Galimam, Hayden Randall, Sophie Heard, Vedant Oze, Mark Robertson

**Affiliations:** 1 Pharmacy, Calvary Mater Hospital, Newcastle, Australia; 2 Royal Prince Alfred Hospital, Australia; 3 Royal North Shore Hospital, Australia; 4 NSW Health Pathology, Gosford Hospital, Australia

## Abstract

**Background:**

Timely microbiological diagnosis is central to antimicrobial stewardship in suspected central nervous system (CNS) infection. In regional settings, access to rapid diagnostics may be limited by off-site testing and reporting delays. We evaluated the association between multiplex polymerase chain reaction (PCR) turnaround time and antimicrobial use and hospital length of stay (LOS).

**Methods:**

We conducted a retrospective cohort study of adult patients (≥18 years) undergoing lumbar puncture for suspected meningitis or encephalitis at two regional Australian hospitals in 2022. Data included time from lumbar puncture to PCR result, antibacterial and antiviral duration and days of therapy (DOT) and LOS. Associations were assessed using Spearman correlation and non-parametric tests.

**Results:**

Longer PCR turnaround time was associated with increased antibacterial (rho = 0.22, *P* = 0.012) and antiviral duration (rho = 0.38, *P* < 0.001). Median antibacterial duration was 2.2 days (range 0.16–10.49) and antiviral duration 2.52 days (range 0.22–21.35). Median LOS was 4.98 days (range 0.38–92.92). Median turnaround time was 44.8 hours (range 15.1–117.22). Overall, 117 patients received antibacterial therapy (median DOT 3 days, range 1–28) and 84 received antiviral therapy (median DOT 3 days, range 1–22). LOS correlated with antimicrobial duration (rho = 0.22, *P* = 0.012).

**Conclusions:**

Delayed PCR reporting was associated with longer antimicrobial exposure and hospital stay, highlighting diagnostic turnaround time as a potential target for antimicrobial stewardship in regional settings.

## Background

Early empiric antimicrobial therapy is critical in suspected central nervous system (CNS) infection to reduce morbidity and mortality. Untreated bacterial meningitis is associated with extremely high mortality and untreated herpes simplex virus encephalitis is associated with severe neurological impairment or death in a substantial proportion of cases.^1,2^ Clinical presentation frequently overlaps between meningitis and encephalitis, with fever, altered mental status and nuchal rigidity commonly observed.^3^

Lumbar puncture and blood cultures are essential investigations and should not delay empiric therapy. While neuroimaging is often performed, it is not routinely required unless features of raised intracranial pressure or immunocompromise are present. Australian Therapeutic Guidelines and NSW Health recommendations advise early broad-spectrum antimicrobial coverage, typically with a third-generation cephalosporin and adjunctive agents based on patient risk factors.^4,5^ Aciclovir is recommended when herpes simplex virus encephalitis is suspected.

Decisions regarding antimicrobial de-escalation remain variable in clinical practice. While guidelines support narrowing therapy when a pathogen is identified, recommendations for cessation when cerebrospinal fluid findings are normal or non-diagnostic are less explicit. Although syndromic PCR assays have short analytical run-times, the clinically relevant turnaround time extends beyond assay run-time to include preanalytical and postanalytical processes such as specimen transport, batching and result reporting.^6^ Delays across this pathway may reduce the practical stewardship benefit of multiplex PCR testing, particularly in regional hospital networks reliant on off-site laboratories.^7^

## Methods

### Study design and setting

This retrospective cohort study included adult patients (≥18 yr) investigated for suspected meningitis or encephalitis at Gosford Hospital (377-bed A1 regional principal referral hospital and regional trauma center with level 5 services) and Wyong Hospital (238-bed B2 regional hospital with level 4 services), both within the Central Coast Local Health District, during 2022.

### Data collection

Collected variables included hospital site, ICU admission, timing of lumbar puncture, timing of multiplex PCR result reporting, duration of antibacterial therapy, duration of antiviral therapy, antibacterial and antiviral days of therapy (DOT) and LOS.

### Turnaround time definition

Turnaround time was defined as the interval from lumbar puncture to multiplex PCR result reporting. Cerebrospinal fluid cell count testing was performed on-site, whereas all multiplex PCR testing was performed off-site at tertiary laboratories.

### Outcomes

The primary outcomes were duration of antibacterial and antiviral therapy and hospital length of stay (LOS). Secondary analyses examined associations between multiplex PCR turnaround time and antimicrobial duration.

### Statistical analysis

Continuous variables were analyzed using Spearman correlation. Group comparisons were performed using Wilcoxon rank sum and Kruskal–Wallis tests, as appropriate. DOT was summarized descriptively and was not included in inferential analyses. Statistical significance was defined as *P* < .05. Multivariate modeling was not undertaken due to incomplete availability of key clinical covariates.

## Results

### Antimicrobial duration and DOT

Median antibacterial duration was 2.2 days (range 0.16–10.49) and median antiviral duration was 2.52 days (range 0.22–21.35). Overall, 117 patients received antibacterial therapy with a median antibacterial DOT of 3 days (range 1–28), and 84 patients received antiviral therapy with a median antiviral DOT of 3 days (range 1–22). Patients admitted to ICU had similar antibacterial (median 2.97 vs 1.96 d) and antiviral durations (median 2.6 vs 2.5 d) compared with ward-managed patients.

### Antimicrobial duration and age

No significant association was observed between age and antibacterial duration (rho = 0.15, *P* = .11) or antiviral duration (rho = 0.06, *P* = .57).

### Hospital site and admitting consultant

No significant differences in antibacterial or antiviral duration were observed between hospital sites or across admitting consultants.

### ICU admission

ICU admission was not significantly associated with antibacterial or antiviral duration.

### Diagnostic turnaround time

Median turnaround time from lumbar puncture to multiplex PCR result reporting was 44.8 hours (range 15.1–117.22; n = 43). In contrast, cerebrospinal fluid cell count results were available rapidly (median 1.15 h; range 0.31–25.35). Longer time from lumbar puncture to multiplex PCR reporting was associated with longer durations of antibacterial therapy (rho = 0.22, *P* = .012) and antiviral therapy (rho = 0.38, *P* < .001).

### Antibiotic and antiviral administration before lumbar puncture

Where documentation permitted, 29 patients received antibacterial therapy and 20 received antiviral therapy prior to lumbar puncture; however, timing relative to lumbar puncture could not be determined in a substantial proportion due to incomplete or inaccurate documentation.

### Length of stay

Hospital LOS was positively associated with both antibacterial and antiviral duration (rho = 0.22, *P* = .012).

## Discussion

In this retrospective regional cohort study, longer multiplex PCR turnaround time was associated with increased antimicrobial duration and longer hospital LOS in patients investigated for suspected CNS infection. The absence of associations with age, admitting consultant, hospital site or ICU admission suggests that antimicrobial duration may be more strongly influenced by system-level diagnostic processes than by individual patient characteristics, location or acuity.

While turnaround time was measured from lumbar puncture to result reporting, this interval reflects a composite diagnostic pathway rather than clinician delay. The implementation of on-site multiplex PCR testing would primarily reduce analytical and postanalytical components of this pathway rather than preanalytical delays.^6^ Prior studies evaluating the BioFire FilmArray meningitis encephalitis panel have reported variable effects on antimicrobial prescribing and hospital utilization, with reductions in antiviral exposure more consistently observed than reductions in antibacterial duration or LOS.

Our findings extend this literature by suggesting that delays in result availability may blunt the expected stewardship benefits of multiplex PCR testing, particularly in regional hospital networks reliant on off-site laboratories.

### Economic implications

Delayed access to diagnostic results has potential economic implications through prolonged antimicrobial exposure and increased hospital LOS. While formal economic evaluation was beyond the scope of this study, antimicrobial duration and occupied bed days are recognized drivers of hospital costs. Improvements in diagnostic turnaround time may therefore support both antimicrobial stewardship objectives and health-service efficiency, particularly in regional hospital settings.

### Limitations

Limitations include the retrospective design, incomplete availability of lumbar puncture timestamps, inability to reliably identify traumatic lumbar punctures, heterogeneity in clinical responses to PCR results, inability to adjust for illness severity or prior antimicrobial exposure and limited postdischarge follow-up. Seasonal variation was not examined as the study objective was to assess diagnostic turnaround time rather than infection incidence patterns. Although an outpatient parenteral antimicrobial therapy (OPAT) service exists in the subject health district, OPAT utilization was not examined as it was outside the scope of this diagnostic turnaround–focused analysis. Analyses were primarily univariable and clinical variables such as cerebrospinal fluid parameters and pathogen identification were not consistently available for multivariate modeling. Turnaround time likely reflects broader system-level processes rather than a direct determinant of prescribing behavior. Attribution of antimicrobial cessation specifically to PCR results was not reliable due to variable documentation and multifactorial clinical decision-making including evolving differential diagnoses and clinician risk tolerance. Finally, the study was conducted within a single regional health district which may limit generalizability to tertiary centers.

## Conclusions

Multiplex PCR turnaround time was associated with antimicrobial duration, DOT and hospital LOS in patients investigated for suspected CNS infection. These findings highlight the stewardship implications of delayed diagnostic reporting in regional hospital settings and support further evaluation of diagnostic pathway optimization.

## Data Availability

Data are available from the corresponding author on reasonable request.
